# Femtosecond laser-assisted LASIK in the SMILE era: a mini review of patient-centered procedure selection

**DOI:** 10.3389/fmed.2026.1908185

**Published:** 2026-07-20

**Authors:** Zitong Yu, Tong Qin

**Affiliations:** 1 Shanghai Bright Eye Hospital, Shanghai, China; 2 Shanghai Jiao Tong University, Shanghai, China

**Keywords:** corneal biomechanics, customized ablation, dry eye, femtosecond laser-assisted LASIK, FS-LASIK, patient selection, refractive surgery, smile

## Abstract

Femtosecond laser-assisted *in situ* keratomileusis (FS-LASIK) remains a mature corneal refractive procedure, but its role is being reshaped by small-incision lenticule extraction (SMILE), transepithelial photorefractive keratectomy, and phakic intraocular lens implantation. This Mini Review proposes a phenotype-centered expert framework, derived from the available literature, for considering when FS-LASIK’s strengths--customized excimer ablation, rapid rehabilitation, enhancement flexibility, presbyopic planning, and hyperopic treatment--may outweigh its flap-related and ocular surface limitations. Evidence is synthesized across visual outcomes, optical quality, ocular surface effects, corneal nerves, biomechanics, regression, epithelial thickness mapping, artificial intelligence-assisted screening, and evolving lenticule platforms. Rather than ranking procedures, the review outlines clinical scenarios in which FS-LASIK, SMILE, TransPRK, or phakic intraocular lens implantation may be appropriate.

## Introduction

LASIK has transformed the surgical correction of myopia and myopic astigmatism. The introduction of femtosecond laser flap creation improved flap predictability and reduced several microkeratome-related complications, giving rise to modern FS-LASIK. The procedure combines femtosecond photodisruption to create a lamellar flap with excimer laser stromal ablation to reshape the cornea; broader reviews of femtosecond laser corneal applications and current refractive surgery paradigms provide the technical background for this evolution (1–3).

The refractive surgery landscape has changed substantially with the widespread adoption of SMILE and the continued development of surface ablation and phakic intraocular lens procedures. Comparative studies increasingly show that procedure choice depends not only on postoperative visual acuity but also on optical quality, ocular surface vulnerability, corneal structural reserve, enhancement expectations, occupational risk, and patient preference (4–11). A useful review should therefore move beyond a broad textbook summary and provide an interpretive framework for choosing among procedures in specific phenotypes.

This Mini Review argues that FS-LASIK remains clinically relevant when positioned as a selective procedure for eyes that are structurally safe and optically likely to benefit from excimer customization. Its unique contribution is a practical phenotype-centered expert synthesis that integrates corneal safety, ocular surface status, refractive phenotype, optical customization needs, presbyopia, hyperopia, and enhancement planning rather than treating FS-LASIK and SMILE as interchangeable competitors.

This topic is timely for three reasons. First, the discussion in clinical practice has shifted from whether laser refractive surgery works to which procedure best matches a given corneal and ocular surface phenotype. Second, newer SMILE platforms and phakic intraocular lens options have raised the standard for how FS-LASIK should justify its role. Third, patient expectations have changed: many candidates ask not only about 20/20 vision but also about night vision, dryness, safety margins, and whether a future enhancement would be possible. A Mini Review format is therefore appropriate because it allows a focused synthesis of recent developments without attempting to duplicate systematic reviews already published on individual outcomes.

## Search strategy and scope

A focused PubMed search was performed for articles published from January 2021 to May 2026, supplemented by earlier landmark studies when they addressed foundational concepts not replaced by recent evidence. Search terms included femtosecond laser-assisted LASIK, FS-LASIK, femtosecond LASIK, SMILE, small-incision lenticule extraction, transepithelial PRK, phakic intraocular lens, dry eye, corneal nerve, corneal biomechanics, ectasia, epithelial thickness mapping, wavefront-guided LASIK, wavefront-optimized LASIK, topography-guided LASIK, ray tracing, presbyopia, hyperopia, enhancement, artificial intelligence, and refractive surgery. Inclusion favored systematic reviews, meta-analyses, prospective or comparative studies, large retrospective series, technology-focused reviews, and clinically relevant studies reporting visual quality, ocular surface, biomechanical, or patient-selection outcomes. Exclusion was applied to non-human studies unless they addressed emerging technology, isolated case reports except for safety signals, and studies without direct relevance to procedure selection. Evidence was synthesized narratively by clinical domain, with recommendations framed as phenotype-based interpretations rather than formal graded guidelines.

## Visual and refractive outcomes

FS-LASIK, SMILE, and surface ablation approaches can all achieve excellent uncorrected distance visual acuity and refractive predictability in appropriately selected patients, although recovery pattern and complication profile differ (12). FS-LASIK often provides faster early visual recovery, while SMILE may have advantages in selected safety domains, particularly flap avoidance and potential ocular surface preservation (10, 11). In high myopia, a meta-analysis found that both SMILE and FS-LASIK were effective, but outcome interpretation should include optical quality, residual stromal reserve, and long-term stability rather than visual acuity alone (13).

Comparative evidence supports a more nuanced procedure-selection model. Studies comparing SMILE, FS-LASIK, TICL, wavefront-optimized LASIK, and topography-guided LASIK indicate that visual acuity may be similarly good across well-selected groups, while astigmatic accuracy, higher-order aberrations, functional optical zone, recovery speed, dry eye, and enhancement options may differ by platform and phenotype (8, 9, 13–15).

## Customized ablation and visual quality

A clinically relevant feature of FS-LASIK is its compatibility with excimer-based customized ablation. Wavefront-guided, wavefront-optimized, topography-guided, and emerging ray-tracing strategies can address refractive error while managing selected optical aberrations. These modalities are not interchangeable: wavefront-guided treatment may be most relevant when reproducible aberrometry identifies clinically meaningful whole-eye higher-order aberrations; wavefront-optimized treatment is commonly used in otherwise regular eyes to preserve corneal asphericity and reduce induced spherical aberration; and topography-guided treatment may be useful when anterior corneal irregularity, asymmetric astigmatism, or decentration dominates the optical problem. Thus, FS-LASIK’s customized options may be most relevant when the surgeon must choose among distinct optical targets rather than simply remove a myopic lenticule.

Visual quality after refractive surgery depends on higher-order aberrations, functional optical zone, centration, contrast sensitivity, glare, halos, corneal densitometry, tear film stability, and epithelial remodeling (16). Reviews on postoperative higher-order aberrations after intraocular lens procedures and systematic reviews on SMILE-related aberrations highlight the increasing importance of optical quality beyond Snellen acuity (14, 17). FS-LASIK may induce spherical aberration or coma, especially in high corrections or decentered treatments; however, customized ablation gives surgeons a tool to mitigate these risks in selected eyes. Long-term observations after SMILE for high myopia also show that higher-order aberrations and microstructural changes remain relevant when comparing modern refractive procedures (18).

## Ocular surface, dry eye, and corneal nerve changes

Dry eye is one of the most clinically important limitations of FS-LASIK. Flap creation transects corneal sensory nerves, reduces corneal sensitivity, and may disrupt the lacrimal functional unit. The magnitude and persistence of postoperative symptoms are influenced by pre-existing dry eye, meibomian gland dysfunction, contact lens history, sex, systemic disease, environment, correction magnitude, and postoperative inflammation.

Recent evidence on tear film stability, tear lipidomics, dendritic cells, corneal nerve morphology, and ocular surface disease after LASIK emphasizes that postoperative dry eye is not a generic complication but a phenotype-dependent risk (6, 7, 19, 20). In counseling, FS-LASIK’s speed and optical flexibility must therefore be weighed against baseline tear dysfunction, meibomian gland disease, contact lens history, sex, systemic disease, and occupational exposure.

Compared with FS-LASIK, SMILE generally involves a smaller incision and may better preserve corneal sub-basal nerves. A systematic review and network meta-analysis reported different nerve effects after SMILE and FS-LASIK, and a recent meta-analysis suggested that keratorefractive lenticule extraction may reduce dry eye outcomes compared with LASIK (21, 22). Clinically, however, ocular surface optimization is essential before any refractive procedure.

## Corneal biomechanics and ectasia risk

FS-LASIK affects corneal biomechanics through both flap creation and stromal tissue removal. Because the anterior stroma contributes substantially to tensile strength, the effect of a flap cannot be judged by residual stromal bed thickness alone. Modern screening should integrate tomography, epithelial thickness mapping, pachymetry, age, refractive magnitude, family history, and, where available, biomechanical metrics.

Epithelial thickness mapping deserves particular attention in the FS-LASIK versus SMILE or PRK decision. Because epithelial remodeling can partially mask stromal irregularity or contact lens-related warpage, ETM may help distinguish a truly suspicious cornea from a borderline topographic pattern that is not ectatic (23, 24). When tomography, pachymetry, biomechanical indices, and ETM are concordantly reassuring, surgeons may be more comfortable considering FS-LASIK for patients who might otherwise be excluded on borderline topography alone. Conversely, an abnormal epithelial pattern should strengthen caution and may shift consideration toward observation, surface ablation, or non-corneal options rather than flap-based surgery.

Meta-analytic evidence suggests that SMILE may preserve certain biomechanical parameters better than FS-LASIK, although measurements vary by device, time point, and study design (15). Matched comparative studies, biomechanical modeling, and studies in post-refractive and keratoconic eyes show that SMILE, FS-LASIK, and PRK have different mechanical consequences and that tomography alone may not fully capture risk (25–28). Post-LASIK ectasia is uncommon but vision-threatening, and risk is increased by abnormal tomography, forme fruste keratoconus, thin cornea, high myopia, excessive ablation depth, low residual stromal bed, high percentage tissue altered, and young age (29).

## Patient selection: FS-LASIK, SMILE, TransPRK, or ICL

The most useful clinical framework is not procedure superiority but patient phenotype. FS-LASIK is attractive for patients with regular corneas, adequate stromal reserve, need for fast recovery, and potential benefit from customized optical correction. It is also practical when enhancement predictability is important. SMILE may be preferable for patients who prioritize flap avoidance, have occupational or sports-related trauma risk, or show increased concern for dry eye and nerve preservation.

TransPRK or other surface ablation procedures remain useful for selected patients with thinner corneas or a reason to avoid flap creation, although recovery is slower and haze prevention must be considered. TICL or other phakic intraocular lens procedures may be preferable for very high myopia, high astigmatism, insufficient corneal reserve, or suspicious tomography. Patient-preference research and comparative outcome studies support transparent counseling and individualized decision-making rather than procedure-centered marketing (8, 30).

**Figure 1:**
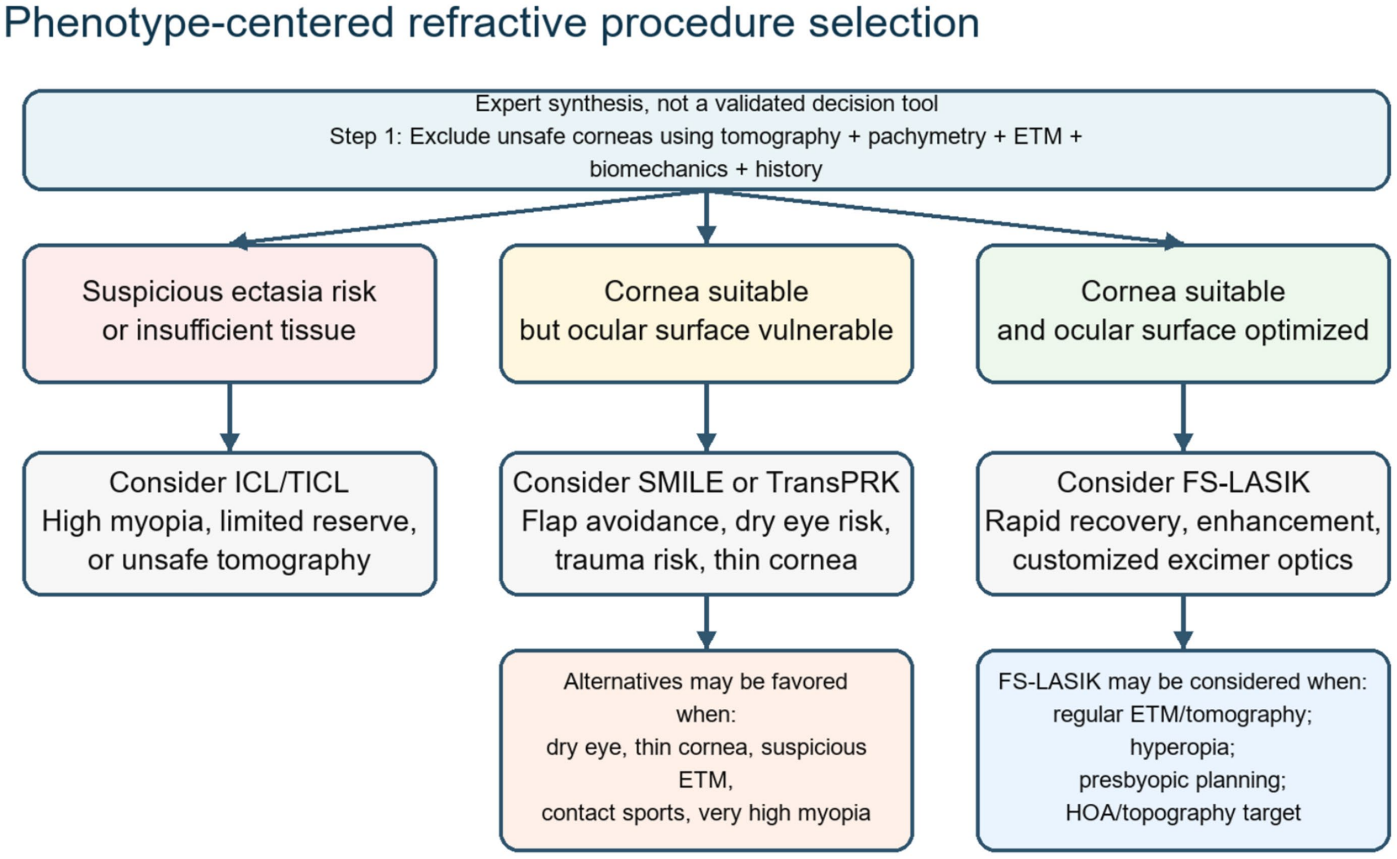
Phenotype-centered decision framework for considering FS-LASIK, SMILE, TransPRK, or ICL/TICL. This algorithm represents an expert synthesis of available evidence and clinical considerations, not a prospectively validated decision tool. The framework starts with corneal safety screening and then integrates ocular surface status, refractive magnitude, optical customization, presbyopic or hyperopic planning, enhancement expectations, and patient-specific priorities. ETM, epithelial thickness mapping; HOA, higher-order aberrations; ICL/TICL, implantable collamer/phakic intraocular lens.

A practical decision sequence is summarized in Figure 1 as an expert synthesis rather than a validated algorithm. First, exclude unsafe corneas using tomography, pachymetry, epithelial mapping, biomechanical information when available, refraction stability, and clinical history. Second, optimize ocular surface disease before final procedure selection. Third, determine whether corneal tissue removal is appropriate for the refractive magnitude; if not, phakic intraocular lens implantation should be considered. Fourth, among corneal procedures, identify the dominant need: optical customization, flap avoidance, surface preservation, enhancement flexibility, presbyopic planning, or hyperopic correction. In this framework, FS-LASIK is not a default procedure but may be considered when excimer customization and rapid rehabilitation are prioritized within a safe corneal profile.

Presbyopic status is another important phenotype, particularly in candidates older than 40 years. For appropriately screened eyes, FS-LASIK can incorporate established presbyopic ablation strategies, including laser blended vision or aspheric micro-monovision approaches, to balance binocular distance, intermediate, and near function (31–33). This flexibility may give FS-LASIK a practical role when the primary goal is simultaneous refractive and presbyopic planning, but evidence remains heterogeneous and counseling must address ocular dominance, adaptation to micro-monovision, mesopic visual quality, reversibility, and the possibility of future lens-based surgery.

Hyperopic refractive error should also be treated as a distinct phenotype rather than as an extension of myopia. In low-to-moderate hyperopia or hyperopic astigmatism with stable refraction, adequate corneal thickness, regular tomography, a healthy ocular surface, and careful angle kappa/centration assessment, FS-LASIK may be considered a strong corneal laser option because hyperopic excimer ablation profiles are clinically established and can be combined with customized or presbyopic planning (32, 34). Current lenticule extraction remains less established for hyperopic correction; therefore, SMILE should not be presented as the default alternative when the main refractive phenotype is hyperopia, while lens-based strategies may become preferable with older age, early lens change, or higher hyperopic demand.

## Regression, enhancement, and complications

Myopic regression after FS-LASIK may reflect epithelial hyperplasia, stromal remodeling, biomechanical forward shift, or initial undercorrection. Higher preoperative myopia, deeper ablation, younger age, and thinner residual stromal bed may increase risk. Recent work on myopic regression after FS-LASIK and SMILE highlights the importance of long-term follow-up and individualized risk assessment (35).

FS-LASIK offers a practical enhancement pathway because flap relifting or surface ablation enhancement is familiar to many surgeons. However, this feature should not obscure its limitations. Potential adverse events include incomplete flap, opaque bubble layer, flap striae, flap displacement, diffuse lamellar keratitis, epithelial ingrowth, infection, central toxic keratopathy, dry eye, glare, halos, undercorrection, overcorrection, regression, and ectasia (36, 37). Accurate centration and cyclotorsion control are especially important for astigmatic correction (5). Newer-generation lenticule extraction platforms, including VisuMax 800, have introduced automated centration and cyclotorsion-compensation utilities, narrowing some historical workflow differences between excimer-based FS-LASIK and lenticule extraction (4).

## Evidence gaps and methodological limitations

Although the refractive surgery literature is large, direct comparison across procedures remains difficult. Studies often differ in laser platform, optical zone, transition zone, attempted correction, flap thickness, cap thickness, lenticule diameter, nomogram, follow-up duration, and enhancement criteria. Dry eye is particularly heterogeneous because investigators use different combinations of symptom scores, tear breakup time, Schirmer testing, ocular surface staining, corneal sensitivity, tear meniscus height, and meibomian gland evaluation. As a result, two studies may both report postoperative dry eye but measure different biological and patient-experienced phenomena.

Another limitation is that many studies emphasize mean postoperative visual acuity and spherical equivalent, which are necessary but insufficient endpoints. Patients frequently care about night driving, glare, halos, fluctuating vision, ocular comfort, time to resume work, fear of flap trauma, and likelihood of future enhancement. These patient-reported outcomes are not consistently captured in FS-LASIK and SMILE comparisons. The recent Frontiers article on preoperative information needs among refractive surgery patients is important because it shows that patient decision-making involves preferences and trade-offs, not only numerical refractive outcomes (30).

Long-term evidence is also uneven. Older studies provide useful durability information, but technology, eye-tracking, nomograms, femtosecond platforms, and ablation algorithms have changed. Conversely, newer customized FS-LASIK and newer SMILE platforms may not yet have enough long-term follow-up. This creates a common evidence gap: the most modern technology has the least long-term evidence, while the longest studies may not fully reflect current practice.

## Future research agenda

Future FS-LASIK research should move toward standardized, phenotype-based reporting. At minimum, studies should report preoperative tomography, epithelial thickness where available, biomechanical indices, optical zone and transition zone, flap thickness, ablation depth, residual stromal bed, percentage tissue altered, ocular surface status, and contact lens history. Without these variables, it is difficult to determine whether postoperative outcomes reflect the procedure itself or the preoperative phenotype of selected patients.

Visual quality outcomes also need harmonization. Future comparative studies should include higher-order aberrations, contrast sensitivity, objective scatter index, functional optical zone, mesopic symptoms, and validated quality-of-vision questionnaires. For dry eye and nerve recovery, corneal sensitivity, *in vivo* confocal microscopy, tear film metrics, and symptom scores should be reported together. This would allow future reviews to connect structural nerve changes with patient-experienced ocular discomfort rather than treating them as separate outcomes.

Emerging concepts are reshaping this decision framework. ETM can reveal epithelial compensation that masks stromal irregularity; biomechanical indices may improve ectasia-risk detection beyond tomography alone; ray-tracing and topography-guided strategies may individualize optical targets; AI-assisted screening may integrate tomography, epithelial mapping, biomechanics, refraction, age, pupil size, dry eye risk, occupational exposure, and patient preference (23, 24, 38). These tools should be viewed as decision support rather than replacements for surgeon judgment, because external validation, explainability, device interoperability, and long-term outcome linkage remain limited.

## Comparison with SMILE and other procedures

The comparison between FS-LASIK and SMILE should not be reduced to a single superiority claim. FS-LASIK is flap-based and excimer-dependent, with high flexibility in optical customization. SMILE is flapless and may be preferred when ocular surface preservation, biomechanical conservation, or avoidance of flap trauma is prioritized. TransPRK and phakic intraocular lenses occupy additional decision spaces rather than serving as simple second-line options: surface ablation may be appropriate for selected thin or flap-avoidant corneas, whereas ICL/TICL may be preferable in very high myopia, limited stromal reserve, or suspicious corneal profiles (see Tables 1, 2).

**Table 1:** Patient-centered comparison of FS-LASIK, SMILE, TransPRK, and ICL/TICL.

Domain	FS-LASIK	SMILE	TransPRK	ICL/TICL
Early recovery	Fast	Fast to moderate	Slower	Fast
Flap-related risk	Present	Absent	Absent	Absent
Dry eye/nerve profile	Higher concern	Often favorable	Variable	No corneal nerve transection
Biomechanical tissue removal	Flap plus ablation	Lenticule extraction	Surface ablation	No corneal tissue removal
Customization	Strong wavefront/topography options	Less mature	Topography-guided possible	Lens-based correction
Best-fit scenario	Regular cornea, fast recovery, customized optics	Flap avoidance, tissue-sparing preference	Thin cornea/flap avoidance	High myopia or limited corneal reserve
Presbyopic planning	Mature blended-vision or aspheric micro-monovision options	Less established for presbyopic ablation	Possible in selected eyes, with slower recovery	Lens-based strategies depend on age and lens status
Hyperopic phenotype	May be considered when low-to-moderate hyperopia/hyperopic astigmatism has regular tomography and suitable centration	Less established for hyperopic correction	Possible in selected eyes but slower recovery/haze considerations	Consider when age, lens status, or corneal reserve favors lens-based planning

**Table 2:** Evidence domains supporting phenotype-centered procedure selection.

Evidence domain	Representative evidence	Clinical implication	Limitations/caveat
Visual recovery and refractive predictability	Comparative FS-LASIK, SMILE, TransPRK, and TICL studies (8, 9, 12, 13, 16)	UDVA may be excellent across procedures; recovery speed and optical quality drive selection	Platform, optical zone, nomogram, and follow-up heterogeneity limit direct ranking
Ocular surface and corneal nerves	Dry eye and nerve analyses after FS-LASIK and SMILE (6, 7, 19–22)	SMILE may be favored in dry eye-prone phenotypes; FS-LASIK requires ocular surface optimization	Dry eye definitions and symptom instruments vary substantially
Biomechanics and ectasia risk	Biomechanical comparisons and ectasia reviews (15, 25–29)	Suspicious tomography, high tissue removal, or low reserve should shift away from FS-LASIK	Device-dependent metrics and rare ectasia outcomes make risk estimation imperfect
Customized excimer optics	Wavefront/topography-guided and optical quality studies (9, 14, 17)	FS-LASIK may be useful when customized optical targeting is the dominant need	Not every eye benefits from customization; centration and tear film remain critical
ETM, AI, and emerging platforms	ETM and AI-assisted screening evidence (4, 23, 24, 38)	Modern screening may refine eligibility and reduce unnecessary exclusion from customized ablation	External validation and long-term outcome linkage remain incomplete
Presbyopia and hyperopia	Presbyopic and hyperopic LASIK evidence (31–34)	FS-LASIK may be considered for selected presbyopic or hyperopic phenotypes	Patient adaptation, lens status, regression, and consensus gaps must be discussed

## Discussion

The central argument of this Mini Review is that FS-LASIK should be understood as a selective procedure with specific strengths rather than as an older competitor to SMILE. Its value lies in rapid recovery, mature enhancement pathways, broad clinical experience, individualized excimer ablation, presbyopic algorithms, and hyperopic correction. These advantages are most meaningful in patients whose corneal structure and ocular surface permit flap-based surgery and whose visual goals may benefit from optical customization.

The main controversy is the trade-off between optical customization and tissue preservation. SMILE may better preserve certain corneal nerves and biomechanical features, but FS-LASIK offers greater flexibility for wavefront- or topography-guided correction. The difference is not merely technological; it affects counseling, expectation management, and long-term satisfaction. Patients should be told that a flapless procedure is not automatically superior and that a flap-based procedure is not automatically inferior.

A balanced appraisal also requires identifying when FS-LASIK should not be favored. SMILE may be preferable when flap avoidance, reduced nerve transection, occupational trauma risk, or biomechanical conservation is the dominant priority. TransPRK may be preferable when a flap is undesirable and surface treatment is acceptable despite slower recovery and haze prophylaxis. ICL/TICL may be preferable when refractive magnitude is high, stromal reserve is inadequate, tomography or ETM is suspicious, or tissue removal would create an unacceptable safety margin. In several of these comparisons, available evidence is limited by non-randomized designs, heterogeneous platforms, evolving nomograms, and short follow-up for newer technologies.

The current evidence base has several limitations. Studies use heterogeneous platforms, nomograms, optical zones, flap thicknesses, dry eye definitions, and follow-up intervals. Patient-reported outcomes are less consistently reported than refractive endpoints. Many comparisons focus on mean visual acuity, which may obscure clinically meaningful differences in night vision, comfort, enhancement likelihood, and satisfaction. Future work should standardize dry eye metrics, corneal sensitivity, epithelial mapping, biomechanical indices, functional optical zone, and validated quality-of-vision instruments.

The practical contribution of this review is therefore a clinician-facing decision framework. A patient with a regular cornea, adequate stromal reserve, healthy ocular surface, hyperopic or presbyopic planning needs, and desire for customized excimer optics may be a reasonable FS-LASIK candidate. A patient with dry eye vulnerability, trauma risk, desire to avoid a flap, suspicious ETM/tomography, thin cornea, or very high myopia may be better served by SMILE, TransPRK, or ICL/TICL. This framing converts the FS-LASIK-versus-SMILE debate into a structured assessment of phenotype, risk tolerance, and optical goals.

## Conclusion

FS-LASIK remains valuable in the SMILE era when selected for the right patient. Its advantages include rapid recovery, predictable refractive outcomes, enhancement feasibility, and compatibility with customized excimer, presbyopic, and hyperopic ablation profiles. Its limitations include dry eye, corneal nerve injury, flap-related complications, biomechanical weakening, and regression. Procedure selection should therefore be phenotype-centered and evidence-aware: FS-LASIK, SMILE, TransPRK, and ICL/TICL should be considered according to corneal structure, ocular surface status, refractive magnitude, epithelial and biomechanical risk, optical demands, age, occupation, and patient expectations.
